# Global Assessment of Extinction Risk to Populations of Sockeye Salmon *Oncorhynchus nerka*

**DOI:** 10.1371/journal.pone.0034065

**Published:** 2012-04-12

**Authors:** Peter S. Rand, Matthew Goslin, Mart R. Gross, James R. Irvine, Xanthippe Augerot, Peter A. McHugh, Victor F. Bugaev

**Affiliations:** 1 Wild Salmon Center, Portland, Oregon, United States of America; 2 Ecotrust, Portland, Oregon, United States of America; 3 Department of Ecology and Evolutionary Biology, University of Toronto, Toronto, Ontario, Canada; 4 Pacific Biological Station, Fisheries and Oceans Canada, Nanaimo, British Columbia, Canada; 5 Pangaea Environmental, LLC, Corvallis, Oregon, United States of America; 6 School of Biological Sciences, University of Canterbury, Christchurch, New Zealand; 7 KamchatNIRO, Pacific Research Institute of Fisheries and Oceanography, Petropavlovsk-Kamchatsky, Russian Federation; Natural History Museum of Denmark, Denmark

## Abstract

**Background:**

Concern about the decline of wild salmon has attracted the attention of the International Union for the Conservation of Nature (IUCN). The IUCN applies quantitative criteria to assess risk of extinction and publishes its results on the Red List of Threatened Species. However, the focus is on the species level and thus may fail to show the risk to populations. The IUCN has adapted their criteria to apply to populations but there exist few examples of this type of assessment. We assessed the status of sockeye salmon Oncorhynchus nerka as a model for application of the IUCN population-level assessments and to provide the first global assessment of the status of an anadromous Pacific salmon.

**Methods/Principal Findings:**

We found from demographic data that the sockeye salmon species is not presently at risk of extinction. We identified 98 independent populations with varying levels of risk within the species' range. Of these, 5 (5%) are already extinct. We analyzed the risk for 62 out of 93 extant populations (67%) and found that 17 of these (27%) are at risk of extinction. The greatest number and concentration of extinct and threatened populations is in the southern part of the North American range, primarily due to overfishing, freshwater habitat loss, dams, hatcheries, and changing ocean conditions.

**Conclusions/Significance:**

Although sockeye salmon are not at risk at the species-level, about one-third of the populations that we analyzed are at risk or already extinct. Without an understanding of risk to biodiversity at the level of populations, the biodiversity loss in salmon would be greatly underrepresented on the Red List. We urge government, conservation organizations, scientists and the public to recognize this limitation of the Red List. We also urge recognition that about one-third of sockeye salmon global population diversity is at risk of extinction or already extinct.

## Introduction

Conservation of biodiversity has become a global concern and the United Nations (UN) and the International Union for the Conservation of Nature (IUCN) are addressing the need to curb the rate of loss of species and the degradation of ecosystems [1]–[3]. The traditional focus has been on determining which species are at risk of extinction, and encouraging safeguards to prevent their loss [4]. Since 1963, species are assessed and their status is documented in the IUCN Red List of Threatened Species™ (hereafter referred to as the Red List, www.iucnredlist.org). However, interest is now growing among conservationists to move from a focus solely at the species level to the identification and protection of populations within species [5], [6]. This change in focus is warranted for several reasons: (1) with several hierarchical levels of biodiversity that might be protected (e.g., genes, individuals, populations, species and communities), it is not evident why assessing species status alone should be chosen; (2) populations often have local genetic adaptations that are critical to the survival of individuals in that region and thus their loss represents an irreplaceable loss within evolutionary time, (3) populations are going extinct at rates faster than that for species, thus the concern for biodiversity loss is happening less at the species level, and (4) the species approach inadvertently allows individual populations to be lost so long as the species is represented in a stable form elsewhere. These concerns suggest an increasing need to understand, track, and respond to threats not just at the species level, but also at the population level. Here we report on the first effort to conduct a global, range-wide status assessment of an anadromous Pacific salmon, sockeye salmon *Oncorhynchus nerka*, using quantitative IUCN criteria at both the species and population levels.

The Endangered Species Act of 1973 and its amendment in 1979 provide recognition of the importance to protect distinct population segments in the United States of America (www.nmfs.noaa.gov/pr/pdfs/laws/esa.pdf). For over a century, many Pacific salmon populations from California through Washington State were depleted or extirpated. In an effort to apply the ESA distinct population segment policy (DPS) to Pacific salmon protection, Robin Waples and the US National Marine Fisheries Service introduced groundbreaking policy to recognize populations within salmon species [7]. Waples' concept of “Evolutionarily Significant Units” (ESUs) below the species level was accepted by the ESA in 1991 as equivalent to “species” and deserving of the full force of protection against extinction [8]. Since then, the Technical Review Teams have been charged with developing recovery plans for threatened and endangered Pacific salmon ESUs [5]. Some of these ESUs are still exploited commercially, but fishing is only permitted if the ‘take’ does not result in harm to the ESU. Similar assessment approaches in which the DPS is akin to the ESU have now been applied to other marine and anadromous species, including steelhead (*O. mykiss*) and Atlantic salmon (*Salmo salar*) [9], [10].

While salmon ESUs have not been formally identified or assessed in the US State of Alaska, the state manages stocks (i.e., typically aggregates of populations) based on the theory of maximum sustainable yield as articulated in their constitution [11]. In some cases, the Board of Fisheries (based on input from the Alaska Department of Fish and Game, ADF&G) has listed stocks as either ‘yield’, ‘management’ or ‘conservation concern’ due to petitions to the Alaska Board of Fisheries. Status of salmon in Alaska is evaluated typically against escapement goals developed by ADF&G [12]. In 2000, the Alaska commercial salmon fisheries became the first salmon fishery to be certified as sustainable against the standards established by the Marine Stewardship Council (MSC, www.msc.org). This evaluation process includes an assessment of the status of stocks. The Alaska salmon fisheries were recertified in 2007. Since this time salmon fisheries have also been MSC certified in Canada and Russia.

In 2003, Canada enacted its Species at Risk Act (SARA, www.sararegistry.gc.ca) to protect biodiversity from extinction. Benefiting from the development of US policy, SARA explicitly recognizes the conservation of populations through its definition of “wildlife species” as “species, sub-species, varieties and geographically and genetically distinct populations.” An independent scientific body, the Committee on the Status of Endangered Wildlife in Canada (COSEWIC, www.cosewic.gc.ca), is authorized to identify biologically distinct populations within species for assessment and makes status recommendations to federal cabinet for protection under SARA. COSEWIC terms these distinct populations “designatable units” (DUs), which are conceptually similar to ESUs in the US. Subsequently, Fisheries and Oceans Canada (DFO) developed a Wild Salmon Policy (WSP) to manage and conserve Pacific salmon in Canada at the population level, which they term salmon Conservation Units [13]–[15]. Thus, conservationists can assess the extinction risk of salmon in both Canada and the USA at the population level for potential protection under endangered species acts [16].

The Red Data Book in Russia originated during the Soviet Era (1961–1964) as an organizational counterpart to the Red List, and in the early 1990s the Russian Federation instituted the Red Data Book into law [17]. The species and subspecies of plants, animals and fungi on the list are protected by the Ministry of Nature. There is a precedent for listing rare species, even without definitive proof of species decline. “Rare” is interpreted either as having a limited geographic range, or being a numerically small population, either of which could heighten a species' vulnerability and provide a rational basis for a precautionary listing. There is currently little precedent in conducting assessments or listing geographically or otherwise distinct populations in the Russian Red Data Book. An important exception is the listing of Kamchatka *O. mykiss* (taxonomically classified in Russia as *Parasalmo mykiss*), a species consisting of sympatric resident rainbow trout and the anadromous form, steelhead [18]. For the commercially valuable species of *Oncorhynchus*, the Federal Fisheries Agency that oversees Pacific salmon in Russia (TINRO) regulates the salmon fishery for maximal yield using escapement goals and monitoring abundance on the spawning grounds [19].

The IUCN Red List is widely recognized as the most comprehensive, objective global approach for evaluating the conservation status of plant and animal species, and plays an increasingly prominent role in guiding activities of government managers, eNGOs and conservation scientists. In 1994 the IUCN introduced a quantitative approach to determine extinction risk that has become the world standard [20]. In 2001, the IUCN adapted its extinction risk criteria to apply at biological levels below the taxonomic level of species [21]. It did this by referring to a species as a “population” or “global population”, and the populations of the species as “subpopulations”. However, few practical examples of IUCN subpopulation assessments exist, particularly in the primary literature [22]. Here we provide a case study of the application of IUCN assessment criteria to a taxonomic species and its constituent populations. Using sockeye salmon *O. nerka*, we apply the Red List criteria for assessing risk to both the sockeye salmon species and its populations throughout its global range including the USA, Canada and Russia. Based on IUCN quantitative criteria, we categorize status and identify leading threats. We discuss the implications of our results at the international and national levels, and provide some recommendations for conservation.

## Materials and Methods

### Description of sockeye salmon

Sockeye salmon were described by Walbaum in 1792. The taxonomic description obscures the large degree of variation in life history among populations within the species. The species occasionally exhibits a freshwater life history form (known as a kokanee); however, due to limited demographic data on this form we focused solely on the predominant anadromous life history form. Sockeye salmon populations are found in continental western USA, north through western Canada and Alaska, and through eastern Russia. Most wild sockeye salmon biomass is found in North America (∼90%), and nearly 50% is in the Bristol Bay region of Alaska.

Anadromous sockeye salmon eggs hatch in gravel nests (redds) in rivers or lakes and typically rear as juveniles for 1 to 3 years in freshwater habitats before migrating to the ocean. Some sockeye salmon assume a river-type life history and rear in a river channel, while others are lake-type and rear in a lake environment. Primary prey during this life history stage include zooplankton and stream invertebrates. Some river-type populations migrate within one to three months following emergence, and these make extensive use of estuaries. Most populations spend 1 to 3 years in offshore feeding areas where they grow to maturity (ca. 50–60 cm total length, 2.5–3.0 kg weight).

Diet in the ocean consists primarily of zooplankton (copepods and euphausiids), but also includes squids and fishes. Natural predators during this oceanic growth period include many other fishes such as salmon sharks *Lamna ditropis*, and Daggertooth *Anotopterus nikparini*. Foraging individuals from many different populations mix to some degree while in the ocean, but at maturity they all migrate back toward their natal freshwater habitat where they spawn and die. While homing in this species is carried out with great precision, some straying may occur. Many individuals are captured by human fishers during the homeward, spawning migration as well as seals, sea lions, killer whales, and in shallow rivers bears, eagles and gulls.

Spawning occurs in late summer and autumn, typically in lake outlets or lake tributary streams or along lake beaches in coarse sediments where subterranean upwelling occurs or among boulders on wave-aerated shores. Sockeye adults typically display bright red bodies and green heads. Males compete with each other for access to females. Females compete with each other for gravel sites where they build nests, deposit eggs (fecundity typically ranges from 2000–5000 eggs), and briefly guard the redd. One consequence of this life history is demographic isolation of spawning populations and thus the opportunity for selection to favor local genetic adaptations. Ecological and molecular studies have confirmed the large degree of genetic differentiation and adaptation among many populations of salmon within the same species [23]. The great diversity of life history characteristics exhibited within the species has been summarized by various authors [24]–[26].

Salmon populations are exposed to many human threats, including commercial and recreational overfishing, mixed-stock fisheries, habitat loss, habitat degradation, negative genetic and ecological interactions with hatchery fish, disease and parasites from fish farms, as well as natural periods of decreased productivity in their ocean environment [27]. These threats operate at both local and broad scales, singularly or in various combinations, and with varying degree of intensity.

### Red List Status Assessment

We generally adhere to Red List terminology but to avoid reader-confusion we substitute the Red List term “subpopulation” with “population” when referring to distinct, genetic groupings within a species, and we use “demes” for “groups” of salmon that are ecologically but not genetically isolated within populations. Our terminology reflects what is commonly used by the U.S. ESA (ESUs) and the Canadian SARA (DUs).

We followed Red List categories and criteria version 3.1 (Table 1, [21]). We used the Red List A2 rather than A1 criterion. The Red List directs the use of A2 for species such as the sockeye salmon where population reduction or its causes may not have ceased or may not be fully understood or may not be reversible. Criteria A1 is applied only in cases when causes of population reduction are clearly reversible and understood and have ceased. We concluded that A1 does not apply to sockeye salmon.

**Table 1:** IUCN Red List criteria applied in the study.

	Threshold by category		
Criterion	CR	EN	VU
A2. Percent decline over last 3 generations (12 years)	80	50	30
B1. Extent of occurrence (km^2^)	100	5,000	20,000
B2. Area of occupancy (km^2^)	10	500	2,000
B2a. Severely fragmented, or number of locations^a^	1	< = 5	< = 10
B2b(iii). Continuing decline in area, extent and/or quality of habitat B2b(v). Continuing decline in number of mature individuals			
D. Absolute abundance	50	500	1,000

a Number of sockeye juvenile nursery lakes and distinct spawning regions within a population.

Quantitative criteria used in the study to determine extinction risk. CR = Critically Endangered, EN = Endangered, VU = Vulnerable.

We applied the Red List B1 and B2 criteria at the species level (including both extent of occurrence and area of occupancy). For the population assessment, we considered the Red List B2a,b(v) criterion, based on area of occupancy, severe fragmentation or the number of extant locations, and the rate of change in the number of mature individuals. In cases where there have been substantial declines in freshwater habitat quality for populations, we evaluated status against Red List B2b(iii) criterion. Since salmon are rarely subjected to “extreme fluctuations” (according to IUCN RL guidelines, variation greater than one order of magnitude either over seasonal or annual cycles), we did not use the Red List B2c criterion. We assessed status based on absolute adult abundance using the thresholds for the Red List D criterion. For all three of these criteria (A, B, and D), we used escapement (reproductively mature individuals that pass or ‘escape’ the fishery and are therefore capable of reproducing) as the measure of population abundance, given that this provides a more direct measure of the number of mature individuals in the population that are capable of reproducing. We use the terms escapement and abundance interchangeably in this paper. We did not consider Red List C and E criteria, which involves projecting habitat conditions and population responses. We felt that this was highly uncertain and beyond the scope of our assessment effort. As per guidelines established by IUCN, the criterion/criteria that returns the greatest risk of extinction is used to characterize status [21]. Below we describe in detail how these criteria were applied in both the species and population level assessments.

The results reported herein reflect a 2011 IUCN amendment to an original assessment completed in 2008 [28]. The amendment differs from the original assessment in two ways. First, the amendment resolved (i.e., split) populations within the Province of British Columbia, resulting in an expansion in the number of assessed populations in this region. Second, we identified the leading threats for each population assessed as threatened in the amendment. We document this process and important differences between the amendment and the original assessment where appropriate below. In the interest of brevity, we do not provide a detailed summary of the results of the original 2008 assessment.

### Species Status Assessment

We computed trends in adult abundance of the global population by estimating the median rate of change across all extant monitoring sites throughout their natural range (data sources and analytical approach described in the next section). The Red List A2 criterion addresses the rate of change in number of adults over a time period of three generations (12 years for sockeye salmon).

Distribution data were essential to estimate area of occupancy and extent of occurrence to be used in evaluation against the Red List B criteria. These criteria address declines in range or fragmentation together with observed declines in number of adults. We used distributional data covering the freshwater breeding range for the species [21], [27], [29]. Distribution for the species was defined for Alaska using the Alaskan Department of Fish and Game Anadromous Waters Catalog [30], for British Columbia using the Department of Fisheries and Oceans' Fisheries Information Summary System [31], for the US Pacific Northwest using Streamnet (Washington, Oregon, and Idaho) [32], and for Russia based on the judgment of local and regional experts, and published accounts. Occurrence was defined at a watershed scale using HYDRO-1K units [33], a globally available GIS basin coverage derived from GTOPO-30 digital elevation model data (30-arcsecond resolution or approximately 1 km^2^ cell size). Extent of occurrence of the species was estimated from the total area of a convex polygon that encompassed all HYDRO1K basins where sockeye salmon were known at one time to have occurred (ca. 150 years before present). Area of occupancy was estimated from the sum of the area of all currently occupied HYDRO1K basins (current refers to approximately 10 years before present). Estimated values were compared to the thresholds defined under Red List criteria B1 and B2 (Table 1).

We estimated a minimum total abundance for the global population by summing all escapement counts at all monitoring sites (average escapement over the last 3 years of counts over the period for which we had data, representing the most recent generation in our time series). This was considered a minimal estimate of global abundance given that much of the data represent index, not total, counts. This was assessed against the Red List D criterion (Table 1).

### Population Status Assessment

#### Population Identification

**Figure 1:**
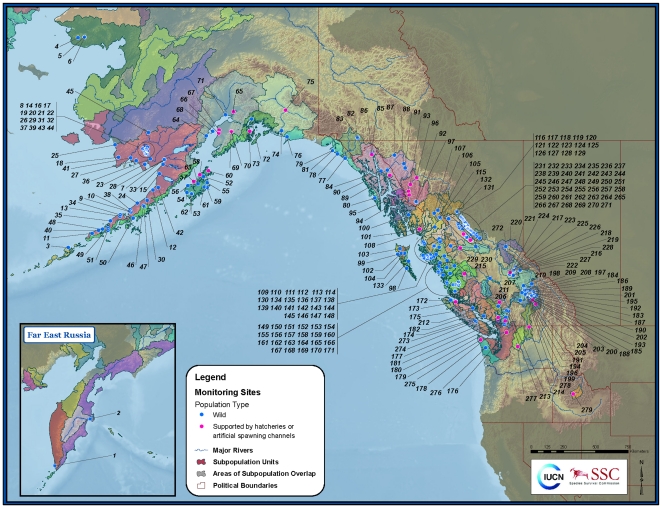
Escapement monitoring sites for sockeye salmon. Escapement monitoring sites for sockeye salmon throughout their natural range in the North Pacific. These sites (N = 279) yielded escapement records spanning at least three generations, or 12 years. Numbers displayed on map correspond to identification numbers for monitoring sites in Tables S1 and S2.

The Red List recognizes populations as geographically or otherwise distinct groups with little demographic or genetic exchange. Populations may, however, contain multiple demes that reproduce at discrete spawning sites. The spawning sites that yielded abundance data for this assessment are referred to here as monitoring sites (Fig. 1). In most cases, we identified populations based on coarsely defined ecoregional groupings and then refined these units based on published genetic data or expert knowledge if these indicated that finer-scale divisions were warranted. We used only natural populations in our assessments; no introduced populations were included.

For our initial population groupings, we used the Level IV Salmon Ecoregions [27], which are delineated according to both the major marine features (oceans, currents, sounds) into which salmon migrate and the major freshwater drainage basins. In order to reduce the candidate set of Level IV Salmon Ecoregions to the subset containing sockeye salmon, we selected only those Ecoregions known to support the species based on expert input. We made finer divisions in large basins in British Columbia (specifically the Skeena and Fraser rivers) based on delineated freshwater zones developed by DFO [14]. This provided a broad spatial habitat template that captured important ecological variables that drive the process of local adaptation in sockeye salmon.

We further subdivided these Ecoregions where significant genetic or population differentiation within Ecoregions was evident. As a first step, we identified the degree of independence among putative populations within each Ecoregion using information on neutral (i.e., non-coding) DNA alleles. While we acknowledge that it is not straightforward to ascribe demographic or geographic distinctness based on genetic distances, we felt it provided a meaningful first step in resolving populations appropriate for status assessment. We acquired data from two sources to identify barriers to gene flow within an Ecoregion. Our primary source was a microsatellite-DNA baseline that represents much of the range of the species [34], [35]. These data include 300 spawning sites across the United States of America, Canada, and Russia (Fig. 1). Our second data source was a matrix of *F_ST_* values (measure of the proportion of total genetic variance contained in a subpopulation, *S*, relative to the total genetic variance of the population, *T*) based on analysis of microsatellite-DNA by the ADF&G Gene Conservation Lab for 55 spawning sites in Bristol Bay, Alaska ([36], Fig. 1). We determined geographic coordinates for all the spawning sites using a combination of agency information, topographic maps, and input from regional biologists. To determine the degree of differentiation among putative populations, we examined the data as a matrix of *F_ST_* values following Cavalli-Sforza chord distances (using PHYLIP v. 3.63). We analyzed these data using a computational geometry that identified both the location and direction of barriers to gene flow (using Monmonier's maximum difference algorithm implemented in BARRIER software [37]).

We used a threshold of 0.04 *F_ST_* across neighboring spawning sites to identify barriers to gene flow within Ecoregions. We arrived at this threshold by applying the Wright-Fisher island model with the following key assumptions: 1) a census population of 6000 individuals (computed as the median population size across all spawning sites in our data base), 2) a N_e_:N_census_ (ratio of the effective population size to the census population) of 0.2 [38], and 3) a threshold exchange rate between sites of <0.5% (corresponding to the Red List guideline of less than 1 migrant exchanged per year). We computed barriers based on a network consisting of Thiessen polygons with each sampled spawning site represented at the center of a polygon. Wherever the threshold *F_ST_* value was reached between two spawning sites, a barrier was identified in the form of an isopleth with the line centered equidistant from the two spawning sites. Once barriers were identified, we derived topographic barriers by aligning the geometric lines developed from the network to drainage boundaries based on digital elevation data or watershed units previously delineated by agencies.

Significant genetic heterogeneity was observed within seven Ecoregions (Transboundary Fjords, Nass-Skeena Estuary, Skeena River, Hecate Strait, Puget Sound, Fraser River, and Columbia River) in British Columbia, Washington, and near the boundary region of Alaska and British Columbia (Fig. 1). We observed much less genetic heterogeneity throughout most of the rest of the species' range, including most of Alaska and the Russian Far East. For example, Bristol Bay sockeye exhibited much less genetic differentiation [36]. We therefore relied on broad Ecoregion boundaries to define populations throughout Alaska and Russia.

Following the release of the original IUCN assessment in 2008, we received suggestions by staff of DFO and the Pacific Salmon Commission (PSC) to resolve populations in British Columbia to better reflect local adaptation and the existence of separately managed salmon populations. Through a series of solicited comments during 2009–2010, we generated a new template that split populations in British Columbia (Table S1, see also [39]). This process resulted in an expansion of the total number of populations across the species' natural range (from 80 populations in the original 2008 assessment to 98 populations in the amendment, Table S1).

#### Application of Red List Criteria

Each population's data were assessed against the Red List criteria described in Table 1. We quantified abundance trends at the level of individual monitoring sites and scaled these results upwards to characterize the status of each population. Data consisted of a series of annual escapement estimates, either in the form of absolute abundance or a standardized index of abundance (e.g., sightings per unit time). The field methods for estimating escapement vary widely, and include aerial and foot surveys, tower, weir, sonar and combinations thereof, as well as different levels of intensity. We obtained escapement data from a variety of sources, and we identified only the most reliable sets based on expert judgement. Data from Russia were obtained primarily from three published sources [40]–[42]. Data from Alaska were obtained primarily from area management reports of the Alaska Department of Fish and Game and the University of Washington Alaska Salmon Program. Data from British Columbia were obtained primarily from DFO. Data from the State of Washington were obtained from a database maintained by the Washington Department of Fish and Wildlife (SASSI data base), the Columbia River Intertribal Fish Commission, and through individual contacts with fisheries biologists in the region. Monitoring sites that yielded data for our assessment are included in Table S2.

Although we identified populations that are enhanced through either hatchery releases or the construction of artificial spawning channels, we did not partition the escapement data among wild-, hatchery-, and spawning channel-origin individuals due to data limitations. In addition, we identified escapement monitoring activities at two discrete levels: Tier 3, which represents an individual spawning site, and Tier 2, which represents an aggregate escapement count that may include numerous spawning sites. Typically Tier 2 escapement enumeration occurs near the river mouth [43]. In general, we assume that little or no in-river take (by humans or other predators) occurs upstream of the point of data collection that might result in an overestimate of abundance of reproducing individuals, particularly in cases where we rely on Tier 2 data. For the purposes of our status assessment, we treated both Tier 2 and Tier 3 populations identically in our analysis. In cases where Tier 3 monitoring was nested within a Tier 2 monitoring effort (e.g., spawning ground visual surveys conducted in a river basin where individuals were counted earlier at a lower, downstream river site), we considered only Tier 3 escapement data as they allow an assessment at finer biological resolution. The Tier 1 level is meant to describe monitoring at a more aggregated level (mixed-stock estimates conducted near the river mouth or along the coastal shelf). While data at this level exist for sockeye salmon (e.g., test fishing, catch-per-unit effort commercial catches), we concluded that these observations are not sufficiently resolved to determine status for this assessment.

For each monitored site, we estimated the average change in adult abundance (i.e., escapement, N) over three generations (i.e., 12 years) using least-squares regression. We used an error-filtering approach to reduce the influence of observer variability on results. Computing trends using raw spawner count data is problematic given the counts represent only a single life stage (spawning adults) and are therefore not a representative sample of the entire population. Further, escapement data are prone to an unknown but high degree of random observer error (e.g., due to incomplete census information, age-structure variation, methodological limitations, and other factors [44]–[46]). Given this, we transformed each data series of length *l* years (where *l* = 15 yrs) to one comprised of 4-year running averages and a length *l*-3 (i.e., 12 yrs). We then estimated the average three-generation change in escapement based on the fitted relationship between log*_e_*(*N*) and year (*t*); the rate of change across a three-generation time window was estimated based on predicted abundance at *t* = 0 and *t* = *t*_max_ [i.e., % change = (*N_t_*_max_−*N_t_*_0_)/*N_t_*_0_*100]. We only considered time series where a minimum of 60% of the years contained observations. We required a minimum of 10 data points in a series (i.e., the series had to be at least 60% complete) to obtain reliable parameter estimates using linear regression [47]. We filled data gaps in incomplete time series using linear interpolation. We relied on the proportional change in escapement over the assessment period to provide a biologically meaningful indication of trend [48].

In order to scale the trends of individual spawning sites (representing individual demes or groups) to the population level, we assigned each population the three-generation change rate (%) equivalent to the median (i.e., 50^th^ percentile) of all monitoring sites for a particular population. For example, if there were six monitoring sites assigned to a given population, we calculated a change rate for each individual site, computed a median change rate across all six sites, and used this median value to represent the overall trend for that particular population. Thus, all monitoring sites contribute equally to the assessment of each population regardless of size or other measure of importance. This approach was meant to underscore the importance of ecological differences, however subtle, across spawning sites in a given population that may be critical for their resilience in the face of future threats [49], [50].

For the population assessment, we used the Red List B2 criterion as it is based upon area of occupancy rather than extent of occurrence. Area of occupancy is more specifically defined by the area of aquatic habitat. Following the Red List recommended procedures, area of occupancy (“the smallest area essential at any life stage to the survival” of existing populations) was estimated for the evaluated population based on a 1 square kilometer grid overlaid on all known nursery lakes and river segments identified as spawning and rearing habitat. Location, as defined by the Red List, is a “geographically or ecologically distinct area in which a single threatening event can rapidly affect all individuals of the taxon present”. In this assessment, we consider each nursery lake and separate, distinct spawning site as locations. Populations qualify for listing under Red List B2 criterion if the area of occupancy falls below the defined thresholds (Table 1) and it satisfies additional criteria (B2a and B2b criteria, see Table 1). As mentioned above, B2c was not considered given sockeye salmon are not prone to “extreme fluctuations” in abundance. It is important to note that at any given point in time, a population of anadromous salmon are distributed in both freshwater and marine habitats, thus a single threatening event impacting freshwater habitat would not directly and immediately harm individuals in the marine phase of their life history. If the threatening event results in permanent alteration of their habitat, than the event could ultimately impact the entire population that is dependent on that habitat to complete their life cycle; thus we feel it is appropriate to apply this in the context of anadromous salmon.

For populations known to have experienced substantial decline in freshwater habitat quality, populations were evaluated against Red List B2a,b(iii) criterion (Table 1). This criterion was applied where there has been extensive hydropower development that has resulted in degraded migratory habitat and altered ecosystem function.

The Red List D criterion addresses population size and the risk of extinction as populations become smaller. We considered the absolute number of mature sockeye adults in our assessment. If the population estimate, determined as an average escapement count over the last generation, fell below the threshold we identified the population as threatened under the appropriate Red List category. It should be noted that abundance estimates were not always a measure of total adult abundance. In many cases (identified by Data Type “I” for Index in Table S2) the abundance value represents a partial population count. In cases where the abundance value for a particular population was low enough to list under the Red List D criterion, we further considered whether the total population might be greater than the Red List threshold values after applying reasonable expansion values in cases where abundance was determined through an index count.

We consider populations to be Extinct based on the Red List definition that there is no reasonable doubt that the last individual has died. This is verified after exhaustive surveys in known and/or expected habitats throughout its historic range have failed to record a single individual [21].

### Retrospective evaluation of Red List A2 Criteria

Given the potential for decadal scale, climate-related shifts in sockeye salmon productivity [51]–[53], it is possible that the use of the Red List A2 criterion does not adequately capture longer-term abundance dynamics and may provide an overly pessimistic picture of current status for particular populations. To assess whether precedent exists for risk-designation reversals and to place our results in a longer term context, we conducted a retrospective evaluation of population status using data from a subset of monitoring sites that have been observed over a long-term basis (i.e., for a minimum of 30 years). In particular, starting with assessment year 1962 (representing the three generations living during the period 1948–1962) and continuing through to the end of the individual time series, we applied Red List A2 criterion (as described above) and quantified the prevalence of differing risk-level assignments at the monitoring site scale (total of 43 sites) for each hypothetical assessment year based on this single criteria. The risk levels were as follows: Vulnerable (VU), Endangered (EN) and Critically Endangered (CR) as defined by the Red List. Though we could not include all assessed monitoring sites in this evaluation (i.e., due to time series length), we incorporated sites from a broad geographic range in North America (19 from Alaska, 24 from British Columbia).

### Identification of Threats

Through a combination of expert input and literature review, we summarized the leading threats to those populations that were assessed as threatened or near threatened. We categorized threats into five general categories (those applicable to this species) as used in the Red List and are applicable to sockeye salmon: Biological Resource Use (fishing), Human intrusions/disturbance (freshwater habitat), Climate/Weather (ocean conditions), System Modification (dams, hatcheries and artificial spawning channels), and Unknown.

## Results

### Species Status

We find that under IUCN assessment criteria, anadromous sockeye salmon are not currently considered to be threatened with extinction at the taxonomic species level and have a status of Least Concern (LC). The median rate of change across the 62 assessed populations indicate an expanding global population (9.0% increase over the past three generations), thus no evidence of risk to the species under Red List A2 criterion. With a calculated geographic range of 11.5 million km^2^, there is no evidence of risk under Red List criterion B1. With a calculated 1.9 million km^2^ of current occupancy (freshwater basin area), and 93 extant populations, there is no evidence of risk under Red List criterion B2. While the historical range of the species has been reduced by approximately 7% due to localized extinction events, the species as a whole is evidently not at risk. With an absolute abundance of the global population in the millions (>19 M mature individuals), there is no evidence of risk under Red List criterion D.

### Population Status

**Figure 2:**
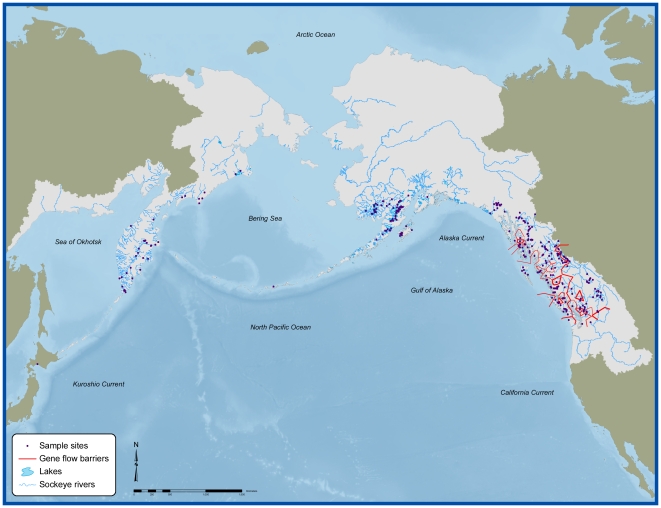
Genetic differentiation among spawning sites of sockeye salmon. Spawning ground sites sampled for two separate microsatellite DNA baselines for sockeye salmon. The presence of significant barriers to gene flow between sites are displayed by red lines (line width scaled to genetic differerentiation). See text for further explanation.

**Figure 3:**
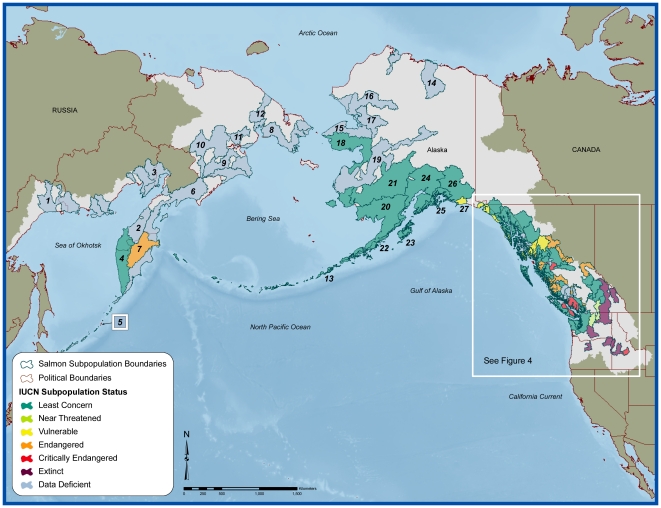
Range-wide map of assessed sockeye salmon and their IUCN status. Numbers displayed on map correspond to identification numbers for sockeye populations listed in Tables S1, S2 and S3.

**Figure 4:**
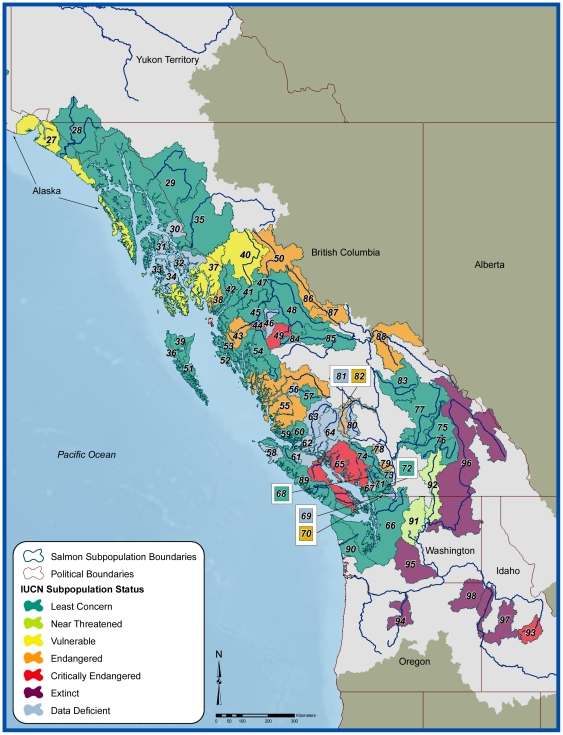
Southeastern range map of assessed sockeye salmon and their IUCN status. Numbers displayed on map correspond to identification numbers for sockeye populations listed in Tables S1, S2 and S3.

We identified 98 distinct populations of sockeye salmon across the natural range of the species (Table S1; Fig. 2). Of these, five populations (5.1%) are now considered extinct. Of the remaining 93 extant populations, 62 (66%) had adequate data for a quantitative assessment of changes in the abundance of mature individuals (termed escapement) across three generations. Our three-generation analyses indicate their abundance trends generally up to 2005 or 2006 (Table S1). The 62 populations differed in number of escapement monitoring sites, ranging from 1 to 38 sites each, with an inter-populational mean of 4.5 monitoring sites and a median of 1.5 monitoring sites per population (Table S1). These sites are listed in Table S2, along with the abundance trend estimated for the past three generations. The abundance trend for the 62 assessed populations, based on the monitoring sites for each population, appear in Table S3. The population summary statistics were compared to the Red List A, B and D criteria described in Table 1. The Red List risk categories assigned to each population are presented in Table S3 and in the form of maps in Figs. 3 and 4. We describe some broad-scale patterns of our results.

Sockeye populations show strong differences in abundance trends across their range (Fig. 3). In southern regions, some populations using lake-river systems in the Hecate Strait-Queen Charlotte Sound, Georgia Basin/Vancouver Island Area, Skeena River and Fraser River, for instance, declined substantially in abundance over the three generations (analyses terminating in 2005 or 2006; Tables S2 and S3, Fig. 4). All five extinct populations were within the Columbia River drainage which is the southern range of the global distribution. By contrast, towards the northern end of their distribution, sockeye were generally characterized by stable-to-increasing trends in adult abundance (Tables S2 and S3, Fig. 3). There were several notable exceptions in the north-to-south risk gradient, including populations in the Columbia River basin and the State of Washington (e.g., populations Wenatchee (SP #91) and Okanogan (SP#92)) although it is important to note that both of these are receiving significant hatchery releases and thus may not accurately reflect wild salmon status. As well, even within large populations where the majority of monitored sites exhibit stable or increasing abundance, some monitoring sites exhibit abundance declines and have remained depressed in recent years (e.g., the Kvichak River population (site #28) in the Southeast Bering (SP #20) has been in a depressed state for several years, whereas other rivers in Bristol Bay have remained productive).

In addition to abundance decline-based criteria, we considered limited geographic range as a factor in extinction risk (Red List B2 criterion, Table 1). A total of 15 populations are threatened (4 as VU, 11 as EN) based on limited area of occupancy or relatively few locations where the taxon was present, and an observed decline in mature adults (Table S3). In addition, two populations (Columbia_Wen, #91, and Columbia_Okan, #92) nearly qualified against Red List B2 criterion on the basis of limited area of occupancy, severe fragmentation and declining quality of migratory habitat (Table 1, Table S3). While numbers of mature adults are not currently declining in these two populations, both have been impacted by extensive hydropower development that fragments and alters the natural ecosystem function of their habitat. Fish in these populations must pass seven and nine dams, respectively, when migrating between the ocean and their spawning grounds. We conclude that these two populations qualify as Near Threatened (NT) because they nearly qualify against Red List B2a,b(iii) criterion (Table S3). For populations NassSkeena_Hugh (#38), Skeena_Alastair (#43), and Fraser_ChilkoS (#82) the Red List B criterion returned the highest threat category among the criteria assessed (Table 3S).

Finally, we considered those populations with abundance low enough to qualify for listing against the Red List D criterion. Only one population, Columbia_Red (#93), qualified as threatened (CR, Table S3). In this case, escapement data were in the form of a dam count, thus the estimate was considered a total count and no abundance expansion was warranted.

In summary, two populations of sockeye salmon are characterized as Near Threatened (NT), three are Vulnerable (VU), 12 are Endangered (EN), and four are Critically Endangered (CR, Figs. 2 and 3, Table S3). Thus 27% of assessed extant populations are at risk based on Red List criteria. A further 32% of all extant populations are Data Deficient, and hence their status is unknown. While all of the countries listed above contained threatened populations, the greatest number and concentration of threatened populations were located in the US Pacific Northwest and the Province of British Columbia. Two populations in the Columbia River, one that spawns in the USA and the other in Canada, show relative stability in their abundance; however, we propose to add them to the Red List as NT given the degree of habitat fragmentation and the degraded quality of their migratory habitat resulting from hydropower development in the region.

### A2 Criteria Retrospective Evaluation

**Figure 5:**
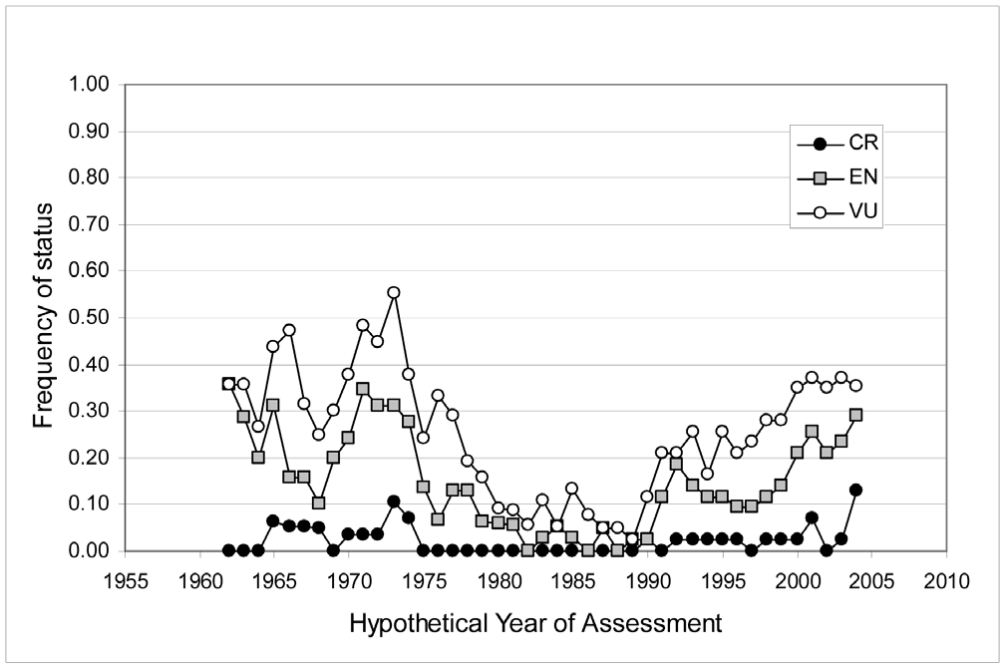
Retrospective analysis to examine effect of temporal trends in populations. Frequency of different threat categories applied using Red List A2 criterion across a series of hypothetical years (1962–2004). CR = Critically Endangered, EN = Endangered, VU = Vulnerable. See text for additional details.

Our retrospective evaluation of Red List A2 criterion revealed that the Red List designations assigned at the monitoring site level are indeed dynamic (Fig. 5). For sockeye in both northern and southern regions, abundance varied in a manner that generated multiple threatened Red List designations for hypothetical assessments conducted between 1962 and the late 1970s, and between 1990 and our current evaluation period. During a period marked by stable-to-increasing abundance, assessments conducted between 1980 and 1990 generated LC designations across most sites. Thus, there appears to be strong coherence between risk characterizations and recognized ocean-climate regimes.

### Threats Identification

It was recognized that certain threats (human disturbance to freshwater habitat, ocean conditions driven by climate patterns) are pervasive, whereas other threats are more localized (fishing, dams, and hatchery development, Table 2). Many sockeye populations are relatively remote, and thus direct threats from land use development are not as pervasive, with some noticeable exceptions (e.g., Cultus Lake in the Lower Fraser Basin, British Columbia, Table 2). Fishing intensity remains a potential threat to some populations, although exploitation rates have been reduced as a precautionary response to declines in abundance over the past decade (Table 2). Fishing impacts on particular populations are in most cases difficult to determine with precision based on the existence of interception and mixed-stock fisheries, and, in some cases, illegal, unreported or unregulated fishing. Dams are considered a threat in the southern portion of the North American range of the species. This threat was identified as the most significant factor in the population extinctions in the Columbia River basin [73]. In addition, negative genetic, ecological and fishery interactions from development of hatcheries and spawning channels are known to be occurring in many sockeye populations (Table 2).

**Table 2:** Identification of leading threats to sockeye salmon populations.

					System Modification			
Population	ID	Biological Resource Use (fishing)	Human intrusions and disturbance (freshwater habitat)	Climate and Weather (ocean conditions)	Dam(s)	Hatchery/Spawning Channel	Unknown	Source^1^ Source^1^
KamRiver	7	X, increasing	X	X			X	[40]–[42], [54], [55]
EGulfAlaska	27	X	X^2^	X			X	[56], [57]
NassSkeena_North	37	X,declining		X		H	X	[56]–[59]
NassSkeena_Hugh	38	X,declining		X		H^3^	X	[56]–[57], [60]
Nass_Upper	40	X		X			X	[61]–[63]
Skeena_Alastair	43	X	X	X			X	[63]–[66]
Skeena_Schul	44	X	X	X			X	[63]–[66]
Skeena_Nan	49	X	X	X			X	[63]–[66]
Skeena_Upper	50	X	X	X			X	[63]–[66]
Hecate_QCS	55	X		X			X	[61]–[63], [67]
PgtGeorgia_Sakinaw	65	X		X		H	X	[61]–[63], [68]
Fraser_CultusL	70	X, declining	X	X		H	X	[69]–[71]
Fraser_Gat	78	X, declining	X	X	X	SC	X	
Fraser_NahatES	79	X, declining	X	X			X	
Fraser_ChilkoS	82	X, declining	X	X			X	
Fraser_EStuart	86		X	X			X	[72]
Fraser_StuartS	87	X, declining	X	X			X	[72]
Fraser_BowronES	88	X, declining	X	X			X	
Columbia_Wen	91		X	X	X	H	X	[73]
Columbia_Okan	92		X	X	X	H	X	[73], [74]
Columbia_Red	93		X	X	X	H	X	[73]

1 Additional input provided by resource agency staff, particularly for populations 70–88 in the Fraser River basin.

2 Uplift from the 1964 earthquake disturbed much of this region, resulting in isostatic glacial rebound that has been recognized as a factor in reduced sockeye escapement, thus some changes to freshwater habitat are not related to human intervention.

3 Hatchery program on Hugh Smith Lake was terminated in 2003.

A list of near threatened and threatened populations of sockeye salmon with identification of key threats specific to each. The sources of information used to identify threats are provided in the final column. Based on information provided by fishery management agencies, fishing pressure on some populations have been intentionally reduced in recent years to encourage recovery (indicated with “X, declining” in the Biological Resource Use column). H = Hatchery, SC = Artificial spawning channel.

## Discussion

This paper provides the first comprehensive effort to assess the global, range-wide status of an anadromous Pacific salmon and its populations using IUCN extinction risk criteria. This effort represents an important departure from more traditional sockeye salmon assessments [75] that impose geo-political or fishery district boundaries as a means of defining assessment units. Further, many assessments evaluate trends at an aggregate level (typically reflected in catch) that can mask trends among the constituent populations (e.g., [75], [76]). While our method of characterizing status involves aggregation across individual monitoring sites, we accounted for the biological, ecological and genetic differences among populations. Further, by assigning a rate of change for each population as the median of the distribution observed across all monitoring sites, all sites contribute equally in our assessment and are not based on relative abundance, productivity or commercial value. This approach detects potential losses in biodiversity from the salmon population ‘portfolio’ [49], [50]. By considering populations based on biological, ecological, and genetic differences our approach provides a more appropriate indicator of the conservation status of the species and its constituent populations. Our analysis represents a step forward in early detection as a means to avoid or reverse erosion of population structure and diversity.

Sockeye salmon are an important commercial fish species and there is ongoing debate about how to best assess its status or “health”. In Table 2 we identify the subpopulations that are currently commercially exploited, and we note the trends in harvest. One form of the debate concerns the relationship between extinction risk criteria (which we have used here) and more traditional fishery management metrics of status, typically expressed as limit reference points [77], [78]. This debate is complicated by different stated goals. Whereas conservationists aim to avoid losses that could lead to extinction, fishery managers aim to avoid losses that could lead to a depressed economic state, where overall productivity is compromised and profit and jobs may be lost. The Red List criteria represent a high tolerance for “false alarms” to minimize “miss rates” and reduce the probability of overlooking populations that may be threatened with extinction whereas fisheries management has low tolerance for false alarms as these can disrupt the fishery and have important economic consequences. In a recent assessment of the Skeena River sockeye fishery, there was an explicit assessment of the trade-off between conservation and fishery exploitation [65]. It was concluded that to comply with Canada's WSP the fisheries targeting sockeye salmon in the Skeena River should be shifted upstream, thereby avoiding catches of less productive populations. We feel this is a useful framework to address this issue, and we encourage application of this approach in other fisheries.

Another dimension to assessing “health” is the degree to which populations are influenced by hatcheries and spawning channels. While some hatcheries clearly have an aim to recover threatened populations (e.g., Redfish Lake in Idaho, USA and Cultus Lake in British Columbia, Canada), there are other programs with an aim to support commercial fisheries (e.g., Copper River sockeye in Alaska, Babine Lake sockeye in British Columbia). While we did not explicitly parse out abundance and trends for wild and hatchery sockeye salmon in our assessment due to data limitations, we acknowledge that there is a growing appreciation of the potential risks posed by hatcheries (e.g., [79], [80]). In our Table 2, we identify the threatened subpopulations that have been, or are currently, influenced by hatcheries or spawning channels, and explicitly identify them as a potential threat to wild sockeye salmon. Clearly, this is a topic that needs considerably more research and conservation attention in the future, particularly as demand for salmon as a desirable source of protein grows and decisions are made about whether to reform or expand hatchery programs.

Salmon productivity has been shown to wax and wane in response to changing ocean temperatures and other large-scale ocean-climate interactions [51]–[53]. Analyzing abundance trends over a three-generation period, as prescribed by IUCN, does not address longer term trends in population dynamics that might result from these climatic regime shifts. An evaluation of 20 different criteria to detect abundance shifts in Fraser River sockeye salmon found that criteria that measured declines from an estimated historical baseline anchored at the beginning of the time series outperformed IUCN criteria based on a three generation time period [81]. The “three generation rule” prescribed by IUCN is perhaps most appropriate in situations where populations do not have natural long-term cycles greater than three generations, or where irreversible threats are occurring to species or populations. When comparable data are available to do so, it can be useful to place the IUCN three-generation criteria within the broader temporal context of longer population trends.

Although our retrospective analysis suggests that current threatened designations based on evaluation against Red List A2 criterion may be short lived for at-risk populations (i.e., reversals may occur without management intervention), three considerations suggest that our general concern is warranted. First, escapements were very low during the three years following the period we analyzed (i.e., 2007–2009). The anomalously high returns of sockeye salmon in the Fraser and Columbia rivers in 2010 remain an enigma. Second, similar to recent findings [72], a shift in conservation status occurred at recognized ocean regime shift boundaries (1977 and 1989), but no change resulted following the shift reported in 1999 [82], [83]. Third, while cyclic highs and lows are a clear feature of historical sockeye production patterns, future ocean-climate conditions remain highly uncertain. This uncertainty translates into an inability to project population trends into the future; global climate change, especially global warming, is forecast to have significant negative impacts on many aspects of salmon life history (e.g., [84]–[87]). There has been a great deal of interest and debate concerning methods of incorporating risks of climate change into the framework for Red List assessments (C. Hilton-Taylor, IUCN Species Programme Office, Cambridge, UK, pers. com). We can expect that new habitat may be created and potentially colonized by sockeye salmon in the Arctic region. But very little is known about this colonization process [88], [89], and it may require proactive management measures to help accommodate range expansion of the species into the Arctic Sea [89].

We do not consider compensatory, density-dependent factors in this assessment. It could be argued that these compensatory factors play a key role in population recovery during periods of reduced overall abundance. This is typically addressed in the form of a stock-recruitment relationship that reflects how productivity (i.e., progeny surviving per spawner) increases at lower spawner abundance. While convincing cases exist of density-dependence in nature [90], populations can also be placed into precarious conditions when driven to low abundance, leading to a depensatory effect [90] or an extinction vortex [91]. Our approach of describing current trends using a linear regression fit to abundance data transformed by a running average provides a straightforward, empirical measure of extinction risk for a population. We feel this is more appropriate than incorporating a complex representation of future trajectories given the uncertainties of compensatory population responses and other factors, particularly climate change. Many of these populations are managed with respect to escapement goals, and hence returns are judged relative to a benchmark. Successful achievement of this objective would be reflected in a relatively stable abundance trajectory and, hence, would be expected to return a non-threatened categorization. Alternatively, fixed exploitation rate approaches could result in cyclic escapement patterns, but the overall trend should indicate stability if fishing pressure is not contributing to an overall decline in abundance. Our analysis does not distinguish the type of fishery or the nature in which fishing is managed. Here we treat ‘take’ as part of environmental variation [92] or as an agent of natural mortality. Addressing fishing dynamics and resulting density-dependent responses is beyond the scope of this assessment effort; however, examining fishing pressure as a potential management ‘lever’ could provide greater insight into the degree of control fishing has on the conservation status of these populations.

Few data were available to assess population viability of sockeye salmon in the Russian Far East, and we document a significant reduction in escapement in recent years for a population within the Kamchatka River basin that warranted a threatened listing. The leading threat recognized for this population is overfishing (Table 2). The situation has been exacerbated by an increase in illegal fishing practices. The mortality of sockeye salmon in the Kamchatka River by poachers is thought to be as high, and perhaps even greater, than the official, legal catch [93]. The economic component of salmon fisheries in the Russian Far East is significant, representing greater than one quarter of the gross regional product in Kamchatka [94]. As economic opportunities in this region have not become diversified, incentives have developed to fish illegally for red caviar as a means to earn money. This is clearly a threat that needs more attention. We encourage the lead agencies in this region to provide more open access to data, and supporting meta-data, for assessment purposes. We also encourage development of new monitoring efforts throughout the region and increased enforcement to reduce poaching [55]. Many populations of river-type sockeye exist in the region, particularly in western Kamchatka, and focused research on these populations may provide insight into their status.

Some of the authors have worked closely with web design professionals to create an interactive, on-line tool (www.stateofthesalmon.org/iucn/new/) to explore the intricacies of this assessment and highlight the important conservation implications of our work with the hope of reaching a wider, more diverse audience. The tool was developed to help facilitate understanding through different learning modalities. The user can explore salmon status through maps, population clusters, historic population phase plots, or simple sorted lists. The tool is designed in a way that the user can easily shift between views, and assemble unique compilations of data for purposes of comparison. The Cluster View provides an engaging new way of displaying individual monitoring sites grouped by population. A simulation using the Historical View provides a unique perspective of the ‘portfolio effect’ as recently described [50] in the context of broader ocean-climate interactions observed over time. While our assessment and this tool only reflects dynamics up to 2006, we feel it would be valuable to update the site periodically and fully re-assess status at 5 year intervals as recommended by IUCN. This could allow us, for the first time, to mark progress on salmon conservation initiatives across this broad geography. Having established our approach and template, these on-line updates and reassessments can be accomplished in a time-efficient manner.

While it is ultimately a societal decision about what actions should be undertaken to protect sockeye salmon populations, we believe that scientists should provide unbiased descriptions of the status of as many populations for which information exists [6] and informed advice about the consequences of actions or inactions [95]. We encourage IUCN assessments to look at units below the taxonomic species level where relevant and the data are available. The analyses contributed here and the resulting awareness of risk status can contribute in a meaningful way to the future conservation of salmon biodiversity.

## Supporting Information

Table S1 **Sockeye salmon populations metadata.**
(XLS)

Table S2 **Abundance trend and data set details for individual sockeye salmon monitoring sites.**
(XLS)

Table S3 **Sockeye salmon population status based on IUCN Red List criteria.**
(XLS)
